# Cariprazine in the management of emotionally unstable personality disorder in female patients: a case series

**DOI:** 10.3389/fpsyt.2024.1421698

**Published:** 2024-07-08

**Authors:** Sofia Pappa, Ellice Caldwell-Dunn, Arturas Kalniunas, Manzar Kamal

**Affiliations:** ^1^ Department of Psychiatry, West London National Health Service (NHS) Trust, London, United Kingdom; ^2^ Department of Brain Sciences, Imperial College London, London, United Kingdom

**Keywords:** cariprazine, partial dopamine agonists, emotionally unstable personality disorder, borderline personality disorder, forensic

## Abstract

**Background:**

Emotionally unstable personality disorder (EUPD) is debilitating psychiatric disorder, particularly common in female and forensic populations. However, appropriate pharmacological treatment to effectively manage symptoms of EUPD remains an unmet clinical need. Dopamine receptor partial agonists (DRPAs), such as aripiprazole, have a favourable tolerability profile and have demonstrated some benefits in targeting symptoms of emotional dysregulation, although, evidence regarding the effects of novel D2/D3 DRPA cariprazine in EUPD patients has been limited.

**Objectives:**

To evaluate the efficacy and tolerability of cariprazine for EUPD in a case series of female forensic inpatients where the diagnosis is more prevalent.

**Methods:**

Demographic and clinical information of the patients were collected from patient electronic records during their admission in a specialized NHS forensic service. Treatment response was measured using the Positive and Negative Syndrome Scale (PANSS) at baseline, 3 and 6 months and Global Clinical Impression Scale (CGI-scores) at baseline and 6 months. Tolerability and BMI, ECG QTc interval and prolactin levels were recorded prior to initiation and at 6 months.

**Results:**

Eight female patients with EUPD (mean age 29.8 years, SD 5.3) were treated with cariprazine (range 3–6mg). Total CGI-scores modestly improved from 5.6 baseline to 5.0 at 6 months. There was a reduction in mean total PANSS scores from baseline to 6 months (92.5, SD 8.1 to 72.4, SD 15.8), general psychopathology (56.1 SD 6.7 to 42.5, SD9.7), positive (21.9 SD 4.6 to 17.1, SD4.8) and negative PANSS scores (14.5 SD 6.3 to 12.8, SD4.6), corresponding to a 21%, 23%, 20% and 3% mean score reduction, respectively. Cariprazine demonstrated a favourable metabolic and hormonal side effect profile with no treatment discontinuation at 6 months follow up.

**Conclusion:**

This is the first case series to evaluate the effectiveness of cariprazine in EUPD. Its efficacy in improving PANSS and CGI-S scores was overall modest and highly variable, reflective of an inherently heterogenous and comorbid patient sample but the benefits on treatment perseverance and tolerability were considerable. Cariprazine may be of particular benefit in EUPD where psychotic symptoms are co-morbid, as an augmentation strategy to clozapine, or where previous antipsychotics have caused metabolic or hormonal side effects.

## Introduction

Emotionally unstable personality disorder (EUPD) is a debilitating psychiatric disorder characterised by a longstanding pattern of affective instability, distorted self-image, impulsivity and unstable interpersonal relationships (1). Individuals with EUPD exhibit marked functional impairment, including high rates of co-morbid psychiatric disorders, substance use, deliberate self-harm (DSH) and suicidal behaviour (2), particularly amongst women, who are three times more likely than men to receive a diagnosis (3). Within forensic cohorts, where it affects up to 35–57% of the population (4), EUPD poses further challenges for the treating clinician, including higher rates of antisocial behaviour with elevated risk of violence towards others, and the necessary emphasis on security, potentially reinforcing serious behavioural problems (4). Furthermore, though EUPD is primarily seen as a disorder of emotional regulation, studies have shown that psychotic symptoms are fairly common, with prevalence ranging from 26% to 54% in clinical populations (5).

Although there are no medications currently formally approved by regulatory agencies for the treatment of EUPD specifically, up to 96% of patients receive at least one psychotropic medication off-label, and polypharmacy is common (6, 7). Pharmacological management strategies have included the use of second-generation antipsychotics (SGAs) (such as risperidone and olanzapine), which are prescribed for up to 70% of inpatients with EUPD (6). Potential target symptoms of SGAs include cognitive-perceptual symptoms, depression, anxiety, anger, impulsivity, paranoia and dissociative behaviour, however high-quality evidence to support use of a single SGA is lacking (8, 9), and long-term use is hindered by adverse side effects (10).

More recently, the so-called ‘third-generation’ of dopamine receptor partial agonists (DRPAs) have gained significant interest in the treatment of psychotic disorders due to their favourable tolerability (i.e. reduced propensity to cause undesirable cardio-metabolic, anticholinergic and hormonal effects frequently encountered with older agents), making them potentially more suitable for long-term use (11). Their pharmacological profile, as evidenced by aripiprazole, may also be beneficial in targeting compulsive traits (12–14). Despite the high prevalence and comorbidity of patients with borderline personality disorders in forensic settings, and the extensive use of atypical antipsychotics and polypharmacy, surprisingly, to date, there is little literature examining their use for EUPD in these populations.

Cariprazine is a novel D2/D3 DRPA, with preferential binding to D3 receptors and a 10-fold higher affinity than for D2 receptors (and with higher affinity than other antipsychotics or dopamine itself) (15), in addition to exerting 5-HT2a antagonism, 5-HT1a partial agonism, and low-moderate affinity for histamine H1, muscarinic and 5-HT2c receptors (15–17). Cariprazine has displayed mood-stabilizing, antipsychotic and pro-cognitive properties in randomised controlled trials examining the spectrum of clinical states of bipolar affective disorder (18), in addition to acute exacerbations, negative symptoms and treatment-resistant cases of schizophrenia (19–23), leading to approval for the treatment of schizophrenia and acute manic or mixed episodes associated with bipolar I disorder in 2015 (24). Cariprazine has additionally shown preliminary evidence for use in resistant bipolar depression (25), as well as an augmentation strategy in major depressive disorder (26), and in those conditions where a previous atypical antipsychotic has failed (27). The evidence around its effectiveness in EUPD has so far been limited to two case reports, with promising results (28, 29). This case series therefore aims to evaluate the efficacy and potential role of cariprazine for the management of EUPD in a group of female forensic inpatients.

## Methods

This case series was conducted in the Women’s Enhanced Medium Secure Services (WEMSS) of West London National Health System (NHS) trust; a large, urban mental health provider in the United Kingdom. WEMSS is a 20-bed medium secure inpatient unit for women who often have multiple psychopathologies and whose behaviour poses a risk of significant harm to themselves or others. The case series consisted of adult female patients who (a) had a diagnosis of EUPD, (b) were WEMSS inpatients during the time of receiving cariprazine, (c) demonstrated adequate mental capacity and (d) were able to provide informed consent. Information was collected as part of a wider NHS service evaluation and was approved by the department for audit and naturalistic research of West London NHS Trust (project number 1775); therefore, it did not require additional research ethics committee approval. Furthermore, informed written consent was not required as part of the specific type of real-world study approval, however, informed verbal consent was obtained from all individuals and documented in electronic records. All data was anonymised to ensure confidentiality and privacy.

Diagnoses of EUPD were made according to ICD-10 criteria by their responsible clinician. WEMSS is a highly specialized forensic unit, and all patients admitted will have completed comprehensive clinical assessment including exclusion of differential diagnoses, and met the threshold for clinically established diagnosis of Emotionally Unstable Personality Disorder according to ICD-10 criteria prior to enrolment. No patient included was pending investigation results (e.g. MRI, EEG) to exclude organic mental disorders. Furthermore, diagnoses are regularly reviewed by their responsible clinician and both primary and secondary diagnoses remained unchanged throughout the trial period. Initiation of cariprazine took place within an inpatient setting subject to independent clinical prescribing decisions made by treating clinicians based on their own medical judgement, and standard of care was unaffected. Medication was administered by clinical staff within the inpatient unit, and the patients were monitored by their respective clinicians involved in their care. Medication titration was started by the patient’s treating team with the lowest dose and adjusting according to symptom response and tolerability.

Demographic and clinical characteristics of the patient population were collected from patient electronic records kept throughout admission to the inpatient unit by their responsible clinical team and psychiatrist. The particular electronic system is called Rio and is commonly used in secondary care services within the National Health Care System in the United Kingdom. Information on previous treatments, reason for switching or augmenting pre-existing medication with cariprazine, tolerability and, where applicable, reasons for discontinuation were also gathered. Treatment response was measured using the Global Clinical Impression Scale (CGI-scores) at baseline and 6 months, and the Positive and Negative Syndrome Scale (PANSS) at baseline, 3 and 6 months at the same time-points as well as subjective patient reports; these were collected retrospectively from electronic records throughout the trial period when reviewed by their responsible clinician. The employment of PANSS was selected according to cariprazine’s main classification as an antipsychotic, but also covering a broad range of psychopathology inclusive of EUPD features. In fact, cariprazine is often used for the management of trans-diagnostic psychotic symptoms, including within the context of EUPD, and therefore PANSS was utilized to capture these effects. As mentioned above, the prevalence of psychotic symptoms in patients with EUPD (particularly in complex presentations) is quite high, either as part of the primary diagnosis or as part of comorbid psychotic disorders. PANSS is additionally inclusive of a broad range of psychopathology (e.g. anxiety, depression, impulse control, hostility) which are frequently present in EUPD patients, and has been used in other patient populations without a schizophrenia spectrum disorder to good effect (30). An interval scale of 1 to 7 was used to measure individual PANSS items. The PANSS score reduction was calculated using the per-protocol population as: (baseline – 6-month score)/baseline x 100. Mean percentage change was derived by considering individual changes for each patient, rather than applying the formula to the mean absolute scores at baseline, 3 and 6 months. Tolerability and safety measures were monitored through clinical interviews and self-reporting throughout the 6-month period, and body mass index (BMI), ECG QTc length and prolactin levels prior to initiating cariprazine and approximately 6 months after initiation.

## Results

Eight female patients with a diagnosis of EUPD were included with a mean (SD) age of 29.8 (5.3) years (range 25–37); the clinical and demographic characteristics of the sample are presented in **Table 1**. All patients were markedly or severely ill (CGI-S 5–6), and had been previously trialled on other antipsychotics. Reasons for initiation of cariprazine included non-response to previous therapy (5/8) and/or side effects of previous antipsychotics (3/8). Cariprazine was used as monotherapy (1/8), in conjunction with antidepressants (3/8) and as an adjunct to antipsychotics (5/8). In one patient, lithium was commenced at 2 months due to emotional lability and elation.

**Table 1:** Demographics and changes in CGI-S and PANSS scores.

Case	Age	Secondary diagnoses	Symptoms at presentation	Previous medication	Concomitant medication	Reason for initiating CPZ	CPZ dose	Clinical outcome	Tolerability	CGI scores baseline	CGI scores 6 months	Total PANSS baseline	Total PANSS 6 months	% reduction total PANSS
**1**	25	Dissocial PD PTSD	Aggression, DSH, emotional dysregulation, impulsivity	Aripiprazole 10mg	Nil	NR	6mg	No appreciable difference in symptomatology or distress.	Good tolerability	Severity: 6	Severity: 6 GI: 4	95	79	17
**2**	23	None	Aggression, DSH, emotional dysregulation, impulsivity, paranoia	Clopixol 400mg IM every 10 days Aripiprazole 15mg	Fluoxetine 60mg Lithium added at 3 months	NR Hyper PRL with clopixol	6mg	Initial reduction in DSH and impulsivity however no difference at 6 months.	Good tolerability	Severity: 5	Severity: 5 GI: 4	79	81	-3
**3**	28	Antisocial traits	Aggression, impulsivity, paranoia	Aripiprazole 10mg	Sertraline 50mg	NR	6mg	Reduction in suspiciousness, paranoia and irritability however continued impulsive aggression.	Good tolerability	Severity: 6	Severity: 5 GI: 3	95	73	23
**4**	35	Dissocial PD	Aggression, DSH, emotional dysregulation, brief periods of psychosis with command auditory hallucinations	Haldol depot 100mg 2 weekly	Venlafaxine 225mg Haldol depot restarted at 4 months	SE	6mg	Initial reduction in aggression and impulsivity, however resurgence in aggressive behaviour at month 4.	Good tolerability	Severity: 6	Severity: 5 GI: 3	92	62	33
**5**	29	Anorexia OCD traits	Aggression, DSH, low mood, anxiety, emotional dysregulation, OCD symptoms, intrusive violent thoughts	Olanzapine depot 300mg fortnightly	Haloperidol 5mg added at 2 months	SE – violent thoughts	6mg Reduced to 3mg due to dizziness	Initial reduction in urges to self-harm however no difference at 6 months.	SE – dizziness at 6mg	Severity: 6	Severity: 5 GI: 3	97	94	3
**6**	26	None	Aggression, DSH, emotional dysregulation, impulsivity	Clopixol 400mg weekly Olanzapine 5mg	Clopixol 300-400mg weekly	SE – hyper PRL, weight gain	6mg Reduced to 3mg – increased ligatures	Increased preoccupation with ligature tying and insertion of objects for sexual satisfaction.	SEs – ligature tying at 6mg	Severity: 5	Severity: 5 GI: 4	85	75	12
**7**	37	Atypical ASD	Aggression, DSH, intrusive violent thoughts	Clozapine 325mg Risperidone 6mg	Clozapine 325mg	Clozapine augmentation	6mg	Initial improvement however at no difference at 6 months.	Good tolerability	Severity: 6	Severity: 6 GI: 4	106	75	29
**8**	35	Paranoid schizophrenia	Aggression, DSH, emotional dysregulation, paranoid delusions, auditory hallucinations	Clozapine 200mg Amisulpride 200mg BD	Clozapine 200mg	Clozapine augmentation	6mg	Marked improvement in aggression, DSH, relationships and psychotic symptoms.	Good tolerability	Severity: 5	Severity: 3 GI: 2	91	40	56
**Mean** **(SD)**	29.8 (5.3)									Severity: 5.6	Severity: 5	92.5 (8.1)	72.4 (15.8)	21 (19)

CPZ, cariprazine, NR, non-response, SE, side effects, PO, per oral, DSH, deliberate self-harm, GI, global improvement, PRL, prolactin.

Overall, most patients (6/8) tolerated cariprazine well without any significant concerns at doses 3–6mg (**Table 2**). Two patients reported adverse effects: one of light-headedness and the other hypersexuality (i.e. increased preoccupation with tying ligatures for sexual satisfaction), both of which resolved when dose was reduced from 6mg to 3mg. Cariprazine displayed favourable effects on metabolic and hormonal parameters; causing modest weight loss in 5/8 patients (2–6kg, mean change in BMI -3.1), and reducing prolactin levels in all patients (where levels were available, mean change -732mIU/L), as well as resolving symptomatic hyperprolactinaemia associated with previous antipsychotic exposure in 3/8 patients. There was minimal effect on the QTc interval (mean change -7ms). No patient discontinued cariprazine within the observed 6-month period, and none were lost to follow-up.

**Table 2:** Tolerability, metabolic, hormonal and cardiac side effect profile.

Case	Tolerability	BMI pre-cariprazine	BMI at 6 months	QTc pre-cariprazine (ms)	QTc post- cariprazine (ms)	Prolactin pre-cariprazine (mIU/L)	Prolactin at 6 months (mIU/L)
**1**	No side effects reported	31	32	482	446	N/A	N/A
**2**	No side effects reported	26	23	455	435	793	393
**3**	No side effects reported	62	56	452	445	964	170
**4**	No side effects reported	43	43	443	440	N/A	N/A
**5**	Light-headedness, dizziness	36	30	406	440	1455	433
**6**	Increased ligature tying and hypersexuality	47	45	447	438	1748	908
**7**	No side effects reported	N/A	N/A	N/A	N/A	430	118
**8**	No side effects reported	36	30	N/A	N/A	1455	433
**Mean change** **(SD)**			-3.1 (3.0)		-7 (23.3)		-732 (306.7)

**Figure 1:**
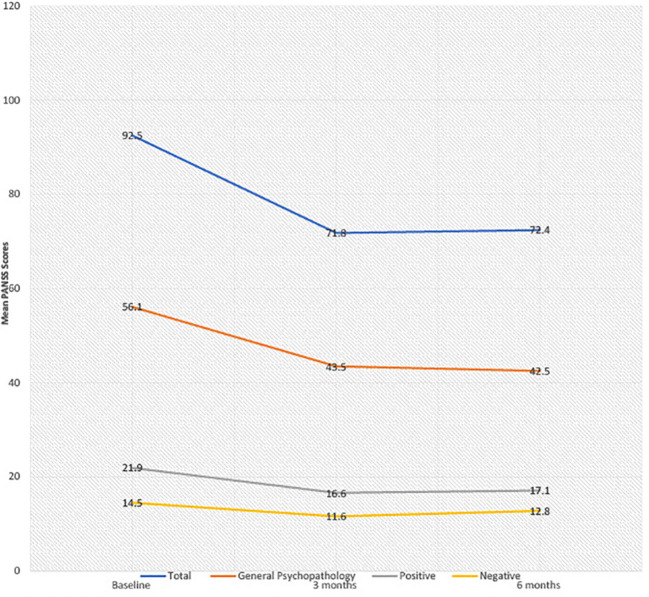
Mean PANSS scores at baseline, 3 months and 6 months.

With regards to treatment efficacy (**Figure 1**), mean CGI-S score modestly improved from 5.6 at baseline to 5.0 at 6 months. There were reductions in mean total PANSS scores from 92.5 to 72.4, general psychopathology scores from 56.1 to 42.5, positive scores from 21.9 to 17.1, and negative scores from 14.5 to 12.8, corresponding to mean percentage decreases of 21%, 23%, 20% and 3%, respectively. In 4/8 patients, initial reductions in PANSS scores at 3 months failed to be sustained at the end of the 6-month period. The reported difference (by patients and responsible clinician) in symptomatology was variable. One patient, where cariprazine was used as an adjunct to clozapine, exhibited marked improvement in symptomatology and distress, including reduced aggression, DSH and psychotic symptoms. Notably, this was the only patient with a comorbid diagnosis of paranoid schizophrenia. Five patients initially reported symptomatic improvement; including reduced aggression, impulsivity and DSH, however these were not sustained at 6 months. One patient exhibited no improvement throughout the 6-month period. In one patient, symptoms of DSH worsened in the context of ligature tying for sexual gratification.

## Discussion

To our knowledge, this is the first case series assessing the effectiveness of cariprazine in forensic patients with EUPD. Overall, cariprazine was well tolerated with all patients persevering with treatment for the follow up duration of 6 months and individual improvements in previous tolerability issues. The effects on CGI-S and PANSS scores were overall modest and highly variable, though, again, individual cases experienced significant improvements. Reductions in total PANSS scores were largely driven by general psychopathology scores, with marginal improvements in low baseline positive and negative scores, and initial reductions in PANSS scores at 3 months were collectively most often not sustained at 6 months.

EUPD is a heterogeneous disorder with considerable symptom overlap with other psychiatric comorbidities (31), reflected in the degree of variability of PANSS score reduction between patients. Furthermore, within EUPD patients, low baseline positive and particularly negative PANSS scores are to be expected, where primary negative symptoms and to an extent psychotic features are not a pronounced problem, explaining modest treatment effect. A collective lack of sustained benefit may be explained by initial placebo effect, inadequate duration trialled or natural fluctuation of mostly complex presentations.

In addition to a favourable metabolic and hormonal profile, Cariprazine demonstrated no adverse effects on cardiac parameters, consistent with previous reports (32–35). Cariprazine’s low affinity for 5-HT2c, H1 and muscarinic receptors reduces the risk of undesirable metabolic, cardiovascular and hyperprolactinaemic effects associated with some of the other antipsychotics (11), having particular benefit where these side effects had proven problematic, for example for those patients with established hyperprolactinaemia at baseline. One patient experienced an increase in ligature tying associated with sexual gratification, potentially as a consequence of previous hyperprolactinaemia and secondarily reduced libido, masking hypersexuality. Comparable to aripiprazole, cariprazine has been associated with, albeit rare, impulse control symptoms including hypersexuality, possibly due to high D3 receptor affinity (36).

In the case exhibiting the greatest improvement in symptomatology, cariprazine was used as an adjunct to clozapine in EUPD with co-morbid paranoid schizophrenia. Within a second case of clozapine augmentation, cariprazine additionally modestly improved suspiciousness and paranoia. Owing to its D2-D3 partial agonism, the antipsychotic properties of cariprazine are well-documented in schizophrenia and acute psychosis (19, 20, 37), and has recently shown to control auditory hallucinations in a case report of EUPD (29). Cariprazine has additionally successfully been used as a clozapine augmentation strategy in schizophrenia in case reports (38–41), and in a recent prospective pilot study (22). Cariprazine therefore potentially merits consideration as a valid treatment option for EUPD with comorbid psychosis, particularly where clozapine has provided only partial therapeutic efficacy and/or in mitigating serious adverse events.

Furthermore, five cases exhibited modest initial improvements in symptoms of impulsivity, aggression and DSH. Although the neurobiology of EUPD is poorly understood, dimensions of emotional dysregulation, impulsivity and DSH are hypothesised to involve both serotonergic and dopaminergic systems (42–44). Cariprazine’s efficacy in affect stabilisation across the spectrum of BPAD states has been hypothetically attributed to partial agonism at presynaptic D3 auto-receptors in the ventral tegmental area, disinhibiting dopamine release in the prefrontal cortex (45). Cariprazine has additionally been noted to reduce hostility in acute exacerbations of schizophrenia (19, 20, 46), and within a case report of EUPD (28). Both 5-HT1a and 5-HT2a receptor sites are implicated in impulsivity, aggression and suicidal behaviour (47, 48), therefore this effect could be attributable to cariprazine’s joint 5-HT1a partial agonism and 5-HT2a antagonism. Cariprazine may therefore be well-positioned to treat both psychotic symptoms and emotional instability.

The main limitations of the study include its small sample size, short period to follow-up and heterogeneity of the data, including potential confounding factors, for example different degree of symptom severity, the presence of multiple psychiatric diagnoses and polypharmacy, and a lack of use of a rating scale specific to EUPD symptoms, e.g. the Zanarini Rating Scale. This was a complex and highly co-morbid sample of patients within a case series and the employment of assessment scales specific to personality disorders would provide a more comprehensive evaluation of personality pathology. Thus, further trials would benefit from the use of specific rating scales, which could aid distinction of the impact of cariprazine from other secondary psychiatric diagnoses. Future trials of cariprazine in community settings where multiple psychiatric comorbidities are less likely to be present may also provide additional clarity on the true effects of cariprazine on EUPD. Furthermore, many patients already had low PANSS scores at baseline, which could have led to an overestimation of the mean percentage reduction.

## Conclusions

Owing to its unique pharmacology, cariprazine has potential in targeting psychotic features, affective dysregulation and hostility frequently seen in EUPD patients. Within this sample, cariprazine demonstrated a modest reduction in CGI and PANSS scores, with some improvement in psychotic symptoms, impulsivity, aggression and DSH within individual cases; however overall improvement was highly variable, due to an inherently complex, heterogenous and comorbid sample of patients. Cariprazine may be of particular benefit in EUPD where psychotic symptoms are co-morbid, as an augmentation strategy to clozapine, or where antipsychotic medication have caused metabolic or hormonal side effects given its favourable tolerability profile. It may be also worth trialing in community patients with less complex presentations of EUPD.

## Data availability statement

The original contributions presented in the study are included in the article/**Supplementary Material**. Further inquiries can be directed to the corresponding author.

## Ethics statement

Ethical approval was not required for the studies involving humans because This study (case series) was approved by the department for audit and naturalistic research of West London NHS Trust (project number 1775) and therefore did not require additional research ethics committee approval or written informed consent. The studies were conducted in accordance with the local legislation and institutional requirements. Written informed consent for participation was not required from the participants or the participants’ legal guardians/next of kin in accordance with the national legislation and institutional requirements because This study (a case series) was approved by the department for audit and naturalistic research of West London NHS Trust (project number 1775) and therefore did not require additional research ethics committee approval or written informed consent.

## Author contributions

SP: Writing – review & editing, Writing – original draft, Visualization, Validation, Supervision, Resources, Project administration, Methodology, Investigation, Formal analysis, Data curation, Conceptualization. ECD: Writing – review & editing, Writing – original draft, Visualization, Project administration, Investigation, Formal analysis, Data curation. AK: Writing – review & editing, Writing – original draft, Project administration, Investigation, Data curation. MK: Writing – review & editing, Writing – original draft, Project administration, Investigation, Data curation.
