# Supplementary material for: Cariprazine in the management of emotionally unstable personality disorder in female patients: a case series
Source: Front Psychiatry. 2024 Jul 8;15:1421698. doi: 10.3389/fpsyt.2024.1421698 (PMC11310661; doi:10.3389/fpsyt.2024.1421698)
Supplement: Supplementary file 1 [file Table_1.docx]

**Supplementary table. Changes in PANSS scores at baseline, 3 and 6 months**

| **Case** | **Baseline total** | **3 month total** | **6 month total** | **Baseline GP** | **3 month GP** | **6 month GP** | **Baseline positive** | **3 month positive** | **6 month positive** | **Baseline negative** | **3 month negative** | **6 month negative** | **% reduction total** | **% reduction GP** | **% reduction**  **positive** | **% reduction negative** |
| --- | --- | --- | --- | --- | --- | --- | --- | --- | --- | --- | --- | --- | --- | --- | --- | --- |
| **1** | 95 | 84 | 79 | 57 | 52 | 49 | 28 | 20 | 20 | 10 | 12 | 10 | 17 | 14 | 29 | 0 |
| **2** | 79 | 55 | 81 | 50 | 37 | 48 | 22 | 11 | 18 | 7 | 7 | 15 | -3 | 4 | 18 | -114 |
| **3** | 95 | 64 | 73 | 64 | 41 | 41 | 15 | 13 | 19 | 16 | 10 | 13 | 23 | 36 | -27 | 19 |
| **4** | 92 | 58 | 62 | 60 | 32 | 36 | 21 | 16 | 17 | 11 | 10 | 9 | 33 | 40 | 19 | 18 |
| **5** | 97 | 87 | 94 | 56 | 54 | 56 | 25 | 24 | 25 | 16 | 9 | 13 | 3 | 0 | 0 | 19 |
| **6** | 85 | 85 | 75 | 43 | 53 | 46 | 27 | 22 | 16 | 15 | 10 | 13 | 12 | -7 | 41 | 13 |
| **7** | 106 | 98 | 75 | 61 | 52 | 40 | 17 | 18 | 13 | 28 | 28 | 22 | 29 | 34 | 24 | 21 |
| **8** | 91 | 43 | 40 | 58 | 27 | 24 | 20 | 9 | 9 | 13 | 7 | 7 | 56 | 56 | 55 | 46 |
| **Mean (SD)** | **92.5**  **(8.1)** | **71.8**  **(19.3)** | **72.4**  **(15.8)** | **56.1**  **(6.7)** | **43.5**  **(10.7)** | **42.5**  **(9.7)** | **21.9**  **(4.6)** | **16.6**  **(5.3)** | **17.1**  **(4.8)** | **14.5**  **(6.3)** | **11.6**  **(6.8)** | **12.8**  **(4.6)** | **21**  **(18.6)** | **23**  **(22.5)** | **20**  **(25.0)** | **3**  **(48.9)** |

GP, general psychopathology
